# Radiation Therapy Infrastructure, Social Vulnerability, and Insurance Architecture Predict Timely Postoperative Radiation Therapy After Brain Tumor Craniotomy

**DOI:** 10.1016/j.adro.2026.102085

**Published:** 2026-05-21

**Authors:** Peter F. Orio, Peter Nakaji, Tim Frandsen, Phillip M. Devlin

**Affiliations:** a Department of Radiation Oncology, Dana-Farber Cancer Institute, Boston, Massachusetts; b Harvard Medical School, Boston, Massachusetts; c John Shufeldt School of Medicine and Advanced Medical Engineering, Arizona State University, Tempe, Arizona; d Department of Neurosurgery, Bob Bové Neuroscience Institute, HonorHealth, Scottsdale, Arizona; e GT Medical Technologies, Tempe, Arizona; f Department of Radiation Oncology, Dana-Farber Brigham Cancer Center, Boston, Massachusetts

## Abstract

**Purpose:**

Timely initiation of radiation therapy (RT) following craniotomy for malignant brain tumors is associated with better clinical outcomes. Despite guideline recommendations and best practices, the timely initiation of postoperative RT remains suboptimal. Our objective was to evaluate how structural, social, and payer-related factors independently and interactively influence RT initiation following craniotomy.

**Methods and Materials:**

We conducted a retrospective, patient-level cohort study using a large, linked, multisource data set that incorporated all-payer claims from 2023, geospatial hospital data, and indicators of community vulnerability. RT initiation was assessed at 42-, 60-, and 90-day postoperative intervals. Primary predictors included hospital referral region-level RT center density, hospital proximity, payer type, and Centers for Disease Control and Prevention/Agency for Toxic Substance and Disease Registry (CDC/ATSDR) Social Vulnerability Index (SVI) domains. Secondary analyses included region and hospital type.

**Results:**

Among 18,885 patients across 548 hospitals, 29.2% initiated RT ≤ 42 days, 45.6% ≤ 60 days, and 48.9% ≤ 90 days. Hospital-level RT initiation ≤42 days ranged from 0% to 87.5%. Hospital referral region per capita RT center density was the strongest structural predictor of RT initiation ≤42 days (adjusted odds ratio [aOR] per IQR, 1.18; 95% CI, 1.11-1.25) and lower odds of noninitiation ≤90 days (aOR, 0.84; 95% CI, 0.78-0.90). Hospital proximity predicted RT initiation ≤42 days (aOR, 1.12; 95% CI, 1.08-1.17) but not ≤90 days. Medicaid coverage predicted lower odds of RT initiation ≤42 days (aOR, 0.58) and higher odds of noninitiation ≤90 days (aOR, 2.29); Medicare coverage predicted lower odds of timely initiation but not of noninitiation (aOR, 0.76). Higher community social vulnerability (Social Vulnerability Index), particularly the share of elderly residents, limited transportation, and housing cost burden, predicted noninitiation, with interaction models showing that hospital density mitigated, but did not eliminate, these disadvantages.

**Conclusions:**

Structural access, socioeconomic disadvantage, and insurance coverage independently and interactively predict the timely initiation of postoperative RT, with greater regional RT capacity improving initiation but not fully mitigating social and payer-related barriers.

## Introduction

Adjuvant radiation therapy (RT) after craniotomy is important for local control and survival in patients with primary and metastatic brain tumors. Timely RT initiation, ideally within 42 days, is supported by studies showing that delays are associated with inferior clinical outcomes.^1^^,^^2^ Even so, the timely initiation of RT varies widely, especially among older, underinsured, and socioeconomically disadvantaged patients.^3^^,^^4^

Prior studies have documented disparities in the receipt of treatment for malignant brain tumors but have not comprehensively examined the initiation of RT within recommended intervals or the system-level factors that influence timely care.^5^ Moreover, registry-based analyses, such as those from the National Cancer Database and Surveillance, Epidemiology, and End Results (SEER),^6^ lack broad measures of social disadvantage alongside structural variables, such as hospital density and geographic access, that reflect the ability to deliver timely RT.

However, delays in postoperative RT may occur at multiple points in the care pathway and result from several patient- and system-level factors. A patient may live near a facility but still experience barriers related to a wide range of factors, such as restrictive payer processes, financial strain, and limited access to transportation. Accordingly, evaluating timely RT initiation requires simultaneous consideration of a broad range of factors. Yet, the extent to which patient-level susceptibility, system-level access barriers, and obstacles related to insurance coverage comprehensively interact to contribute to low rates of RT initiation is not well understood, despite established guideline-based recommendations for initiation of postoperative RT.^7^

Using a large, multisource data set linking payer claims to county, ZIP, and hospital referral region (HRR)-level indicators (including broad socioeconomic risk indicators, census-derived measures, geospatial data, and the International Atomic Energy Agency’s Directory of Radiotherapy Centers), this study integrates multiple dimensions to examine how structural, social, and payer factors interact to influence timely RT initiation after a craniotomy.

## Methods and Materials

We conducted a retrospective analysis using a data set created by linking multiple sources. Claims data were obtained from the Definitive Healthcare Atlas all-payer database for calendar year 2023, which contains over 12 billion medical claims representing nearly 330 million covered lives. Broad risk indicators (structural and socioeconomic) were from the U.S. Census Bureau’s American Community Survey (ACS) and the Centers for Disease Control and Prevention/Agency for Toxic Substance and Disease Registry (CDC/ATSDR) Social Vulnerability Index (SVI; 2022) databases. HRR assignments were obtained from the Dartmouth Atlas of Health Care Zip-to-HRR crosswalk, and RT-capable centers were identified using the International Atomic Energy Agency’s Directory of Radiotherapy Centers. Lastly, geospatial coordinates were obtained directly from the Definitive Healthcare Atlas database.

Adult patients (≥18 years) who underwent a craniotomy for malignant brain tumors from January 1, 2023, to December 31, 2023, were included. Patients were identified using International Classification of Disease, 10th Revision, Procedure Coding System (ICD-10-PCS) codes for craniotomy, and International Classification of Disease, 10th Revision, Clinical Modification (ICD-10-CM) diagnosis codes for malignant neoplasm of the brain (primary or metastatic), based on the official FY2023 ICD-10-CM and ICD-10-PCS coding definitions.^8^^,^^9^ Those who died or entered hospice within 90 days were excluded. The initiation of RT delivery was defined as the earliest RT delivery date recorded for a claim within 90 days of the surgery. Patients were required to have observable claims for 90 days, as determined by payer linkage to the all-payer file. Billing and reimbursement codes used to select eligible patients are provided in Table 1.

**Table 1:** Codes used to select eligible patients

Craniotomy ICD-10-PCS codes	Malignant brain tumor ICD-10-CM codes	RT treatment delivery CPT codes
00B70ZZ	C70.0	61796
00B00ZZ	C71.0-71.9	61798
00BC0ZZ	C79.31-79.32	77402-77412
00B60ZZ	-	77371-77373
00B10ZZ	-	77385-77386
00B20ZZ	-	77520-77525
		G6015

*Abbreviations:* RT = radiation therapy; ICD-10-PCS = International Classification of Disease, 10th Revision, Procedure Coding System; ICD-10-CM = International Classification of Disease, 10th Revision, Clinical Modification; CPT = Current Procedural Terminology.

Patients with RT delivery claims before the craniotomy were excluded from the analytical cohort because the study endpoint was postoperative RT initiation. To assess the potential impact of including patients who received preoperative RT, we separately identified patients with RT delivery claims within 30 days of surgery. This descriptive look-back analysis was not included in the primary models because preoperative RT represents a distinct treatment sequence in which patients are not at risk of the same postoperative initiation endpoint.

RT initiation was assessed at 42-, 60-, and 90-day postoperative intervals. RT initiation ≤42 days was considered timely initiation; ≤60 days was descriptive only for characterizing intermediate initiation; and no initiation ≤90 days was defined as noninitiation. These intervals were patient-level binary endpoints rather than time-to-event measurements.

Candidate predictors included hospital type (academic vs community, derived from the Centers for Medicare and Medicaid Services (CMS) teaching hospital list) and measures of local access. Population density was calculated as county population divided by land area using ACS data, and then log-transformed to reduce skewness. Hospital proximity was characterized by the nearest-neighbor distance and the number of hospitals within 10, 15, and 25 miles of the index hospital (the hospital where the craniotomy was performed), with the latter used as a sensitivity exposure. HRR-level capacity was measured by 2 metrics: per capita RT center density (centers per 100,000 residents) and HRR per area density (centers per 100 square miles, used as a sensitivity exposure).

Additional variables included patient-level payer type (commercial, Medicare, and Medicaid) and community-level vulnerability measures. Social vulnerability was measured using the 2022 CDC/ATSDR SVI, derived from ACS data and comprising 16 indicators grouped into 4 themes: (1) socioeconomic status; (2) household characteristics; (3) racial and ethnic minority status; and (4) housing type/transportation. Socioeconomic status (theme 1) includes measures such as poverty, unemployment, education, and housing cost burden. Household characteristics (theme 2) include measures such as age, disability status, single-parent household status, and English-language proficiency. Racial and ethnic minority status (theme 3) captures the proportion of residents who identify as members of racial or ethnic minority groups. Housing type/transportation (theme 4) includes measures such as multiunit housing, mobile homes, crowding, and households without vehicle access.

In addition to the broad SVI themes, we evaluated individual SVI indicators that represented plausible barriers to postoperative RT initiation, including age, racial and ethnic minority status, vehicle access, and housing cost burden. Housing cost burden is defined as the proportion of households spending ≥30% of their income on housing. The transportation-related SVI measure used in this analysis reflects the proportion of households without access to a vehicle and does not directly measure the availability of public transportation or the walkability of the area.

Analyses were performed at the patient level, treating hospitals as clustering units. Descriptive summaries of timely RT initiation and noninitiation were expressed as proportions of patients who initiated RT within each time interval. Inferential analyses focused on 2 primary endpoints (RT initiation ≤42 days and nonnitiation ≤90 days). Proportions were compared by payer type, census region/division, and HRR density quartiles using χ² tests of independence.

Independent predictors of RT initiation were evaluated using mixed-effects multivariable logistic regression models that included a hospital random intercept to account for clustering of patients within hospitals. Regional fixed effects were included, and HRR fixed effects were tested in sensitivity analyses. Interaction terms between hospital density and SVI measures were tested to evaluate compounded disadvantage. Sensitivity analyses stratified results by hospital type and disease category (primary vs secondary/metastatic). Adjusted odds ratios (aORs) with 95% CIs are reported. A 2-sided *P* < .05 was considered statistically significant. Tables summarizing RT initiation report the cumulative portion initiated at 42, 60, and 90 days. For regression models, outcomes were RT initiation ≤42 days versus >42 days, and noninitiation ≤90 days versus initiation ≤90 days. Analyses were performed with SPSS v30.0 (IBM Corporation).

This study used deidentified, commercially available data and was determined to be exempt from human subjects review under 45 CFR 46.104(d)(4).

## Results

Among 18,885 patients across 548 hospitals, 29.2% initiated RT ≤ 42 days, 45.6% ≤ 60 days (descriptive only), and 48.9% ≤ 90 days (51.1% did not initiate RT ≤ 90 days). Hospital-level RT initiation ≤42 days ranged from 0% to 87.5%.

Comparing RT initiation rates by payer type, patients with commercial coverage had the highest rates at both the ≤42- and ≤90-day endpoints, whereas those with Medicaid had the lowest. χ² test results indicated that unadjusted RT initiation by payer type was significantly different (Table 2). In adjusted models using commercial insurance as the reference group, payer type was a strong independent predictor of RT initiation. Medicaid and Medicare coverage were both independently associated with lower odds of RT initiation ≤42 days (aOR, 0.58; 95% CI, 0.42-0.75; *P* < .001 and aOR, 0.76; 95% CI, 0.70-0.83; *P* < .001, respectively). However, in the ≤90-day model, Medicare coverage was not predictive of noninitiation (aOR, 0.93; 95% CI, 0.60-1.06; *P* = .33), whereas Medicaid was highly predictive of noninitiation (aOR, 2.29; 95% CI, 1.10-3.95; *P* = .02) (Table 3).

**Table 2:** Initiation by payer type

Payer type	≤42 d, n (%)	≤60 d, n (%)	≤90 d, n (%)
Commercial	2584 (33.4)	3904 (50.4)	4167 (53.8)
Medicare	2269 (26.7)	3768 (44.3)	4072 (47.9)
Medicaid	667 (24.9)	949 (35.9)	995 (38.1)
Total	5520 (29.2)	8621 (45.6)	9234 (48.9)
χ² test (*df*)	310.6 (2)	-	290.3 (2)
*P* value	<.001	-	<.001

Comparison of radiation therapy initiation rates by payer type. Patients with commercial coverage had the highest radiation therapy initiation rates at all time points, while those with Medicaid had the lowest. Values are cumulative percentages initiated at x days. X^2^ test results indicate that unadjusted initiation by payer type was significantly different at both time points. Values at ≤60 days are descriptive only; inferential tests and models were restricted to ≤42 days, and noninitiation to ≤90 days.

**Table 3:** Independent predictors of initiation

Predictor	≤42 d aOR (95% CI); *P* value	No initiation ≤ 90 d aOR (95% CI); *P* value
Hospital and regional factors		
Hospitals within 10 mi	1.12 (1.08-1.17); <.001	0.99 (0.89-1.08); .48
HRR per capita RT center density (per IQR)	1.18 (1.11-1.25); <.001	0.84 (0.78-0.90); <.001
Hospital type (academic vs community)	1.03 (0.97-1.09); .18	0.96 (0.91-1.03); .44
Social vulnerability factors		
SVI: ≥65 y	0.95 (0.91-0.98); .04	1.06 (1.02-1.11); .005
SVI: access to transportation	0.82 (0.78-0.88); <.001	1.12 (1.07-1.17); <.001
SVI: minority	0.79 (0.67-0.93); .005	1.28 (1.16-1.41); <.001
Housing cost burden	0.87 (0.78-0.97); .02	1.14 (1.02-1.28); .02
Insurance coverage		
Medicare insurance	0.76 (0.70-0.83); <.001	0.93 (0.60-1.06); .33
Medicaid insurance	0.58 (0.42-0.75); <.001	2.29 (1.10-3.95); .02

Multivariate regression results expressed as adjusted odds ratios (aOR), 95% confidence intervals (CI), and p-values for independent predictors. HRR per capita RT center density is defined as the number of RT-capable centers per 100,000 residents within each HRR; IQR = 0.45 to 0.85 centers/100,000 residents. aORs are per IQR increase. Commercial insurance is the reference for payer comparisons.

*Abbreviations:* aOR = adjusted odds ratio; HRR = hospital referral region; RT = radiation therapy; SVI = Social Vulnerability Index.

After adjusting for HRR density and SVI domains, hospital type was not independently associated with either endpoint (Table 3). In unadjusted analyses, academic/teaching hospitals demonstrated significantly lower ≤42-day RT initiation rates than community hospitals (Table 4; *P* < .001), but this association was no longer significant after adjusting for per capita HRR capacity, proximal access, and SVI (*P* = .18). This indicates that reports of lower RT initiation rates observed in academic settings may be attenuated by geographic and social contextual factors rather than by intrinsic differences in hospital type.

**Table 4:** Initiation by hospital type

Hospital type	≤42 d, %	≤60 d, %	≤90 d, %
Academic	27.8 ± 28.8	45.9 ± 27.9	49.1 ± 29.6
Community	30.1 ± 33.1	45.4 ± 32.8	48.8 ± 33.4
*P* value (academic vs community)	<.001	-	.50

Comparison of radiation therapy initiation rates by hospital type. In the unadjusted analysis, academic/teaching hospitals had significantly lower radiation therapy initiation rates than community hospitals at 42 days, but the difference was no longer significant at 90 days. Values at ≤60 days are descriptive only; hypothesis tests and models were restricted to ≤42 days, and noninitiation to ≤90 days. Data are presented as mean ± SD.

As shown in Table 3, higher per capita HRR density was predictive of ≤42-day RT initiation (aOR, 1.18; 95% CI, 1.11-1.25; *P* < .001) and lower odds of noninitiation ≤90 days (aOR, 0.84; 95% CI, 0.78-0.90; *P* < .001). Tests for nonlinearity using spline terms were not significant (global spline *P* = .12). Substituting per area HRR density (centers per 100 square miles) for per capita density yielded smaller but directionally similar statistically significant associations (≤42 days aOR per IQR, 1.09; 95% CI, 1.01-1.17; noninitiation ≤90 days aOR per IQR, 0.91; 95% CI, 0.84-0.99).

In addition, the number of hospitals within 10 miles was independently associated with higher≤ 42-day RT initiation rates, even after accounting for HRR-level hospital density (aOR, 1.12; 95% CI, 1.08-1.17; *P* < .001). However, this hospital distance measure was not associated with lower odds of noninitiation in the 90-day model (aOR, 0.99; 95% CI, 0.89-1.08; *P* = .48) (Table 3). After adjusting for hospital density, population density was not independently associated with either endpoint (*P* > .20), suggesting that urbanicity and population density effects were captured by these continuous measures.

Multiple SVI variables were shown to be independent predictors of lower RT initiation rates, specifically the share of elderly residents, access to transportation, minority status, and housing cost burden (Table 3). Interaction analysis indicated that this disadvantage was amplified by a lack of access to transportation (aOR, 0.94; 95% CI, 0.89-0.99; *P* = .03) and a high housing cost burden (aOR, 0.63; 95% CI, 0.60-0.66; *P* < .001). Limited transportation access was an independent predictor of lower ≤42-day RT initiation rates (aOR, 0.82; 95% CI, 0.78-0.88; *P* < .001). However, this effect was attenuated in high-density regions (interaction odds ratio, 1.08; 95% CI, 1.02-1.14; *P* = .01) (Table 5), indicating that greater hospital density mitigates but does not eliminate the adverse impact of poor transportation, likely reflecting closer proximity to RT facilities and the availability of public transportation options.

**Table 5:** Interaction effects

Interaction term	OR (95% CI)	*P* value
Age ≥ 65 y + transportation	0.94 (0.89-0.99)	.03
Age ≥ 65 y + housing cost burden	0.63 (0.60-0.66)	<.001
Transportation + hospital density	1.08 (1.02-1.14)	.01

Interaction terms were used to evaluate potential effect modification and to determine whether the influence of social vulnerability varied by urbanicity or structural capacity. *P* < .05 is considered statistically significant.

*Abbreviations:* OR = odds ratio.

Higher housing cost burden and SVI domains related to racial and ethnic minority population share, older age, and limited access to transportation were associated with lower RT initiation rates, independent of population density. These factors had consistent negative effects in both rural and urban settings and remained significant after adjustment for hospital type, regional capacity, and payer mix. These patterns persisted in fully adjusted models, highlighting how social and economic conditions affect real-world treatment delivery. Together with population HRR density interactions, these results suggest that RT initiation rates are higher in urban settings, but social disadvantage continues to create barriers to care, even in resource-rich settings. All statistically significant independent predictors of RT initiation are provided in Table 3, and statistically significant interaction effects are provided in Table 5.

When hospitals were grouped by census region and division, RT initiation rates varied significantly in the unadjusted analyses. Hospitals in the Northeast and Midwest generally had higher RT initiation rates, whereas those in the South and West had lower initiation rates (χ² test, *P* < .001). At the division level, hospitals in New England and the Middle Atlantic had the highest RT initiation rates, whereas those in the Southern divisions had the lowest initiation rates (χ² test, *P* < .001; Table 6). In the adjusted models, these differences were not independently predictive, suggesting that structural and social factors underlie the observed geographic patterns.

**Table 6:** Geographic differences in initiation

		Initiation ≤ 42 d		Initiation ≤ 60 d		Initiation ≤ 90 d	
Census division	n	Mean, %	SD, %	Mean, %	SD, %	Mean, %	SD, %
New England	976	36.5	20.6	56.1	21.6	59.3	21.5
Middle Atlantic	2321	41.2	15.7	59.2	18.3	61.1	17.0
East North Central	3138	32.5	15.5	47.9	17.3	52.9	18.4
West North Central	1408	30.3	15.1	47.0	16.2	49.9	17.6
South Atlantic	3955	26.0	15.6	44.2	18.3	46.7	19.7
East South Central	1007	23.2	15.3	37.1	16.9	39.8	18.0
West South Central	2557	21.5	11.2	33.3	13.4	37.7	13.7
Mountain	1281	25.1	13.3	40.3	15.8	44.9	17.2
Pacific	2242	27.7	14.2	46.2	16.4	48.5	18.3
χ² test; *P* value		208.4; .001				270.4; .001	

Census region	n	Mean, %	SD, %	Mean, %	SD, %	Mean, %	SD, %
Northeast	3298	39.8	17.1	58.3	19.2	60.6	18.4
Midwest	4546	31.8	15.3	47.6	16.9	52.0	18.1
South	7518	24.1	14.2	39.5	16.6	42.7	17.7
West	3523	26.8	13.8	44.1	16.1	47.2	17.9
χ² test; *P* value		181.7; .001				221.5; .001	

Radiation therapy initiation rates across census regions and divisions. Values represent hospital-level initiation proportions. X^2^ test results demonstrate statistically significant differences in radiation therapy initiation rates between regions and divisions.

When evaluating preoperative RT use among patients excluded from the primary analytical cohort because they had received RT prior to surgery, 320 had RT delivery claims ≤ 30 days of surgery. Because this preoperative RT group was small relative to the 18,885 patients included in the postoperative RT cohort, adding these patients to the analytical cohort and counting them as having received RT ≤ 42 days did not change the study findings, including the associations with payer type, HRR RT center density, transportation access, and housing cost burden.

## Discussion

This study demonstrates that postoperative RT initiation after craniotomy is influenced by intersecting structural, social, and insurance-based determinants. HRR per capita RT center capacity, defined as the supply of RT-capable centers relative to the HRR population, was the most consistent structural predictor of timely RT initiation. RT center density (centers per 100 square miles) showed smaller but significant effects in the sensitivity analyses. These HRR center capacity effects persisted even after adjusting for hospital type, social and community factors, and region. This is consistent with prior literature linking radiation infrastructure distribution to oncologic outcomes and broader calls for multilevel approaches to achieving equitable cancer care.^10^, ^11^, ^12^

We chose ≤42 days as the RT initiation cut-point because multiple studies indicate that delays beyond 42 days may negatively affect survival outcomes. Moreover, it is consistent with the American Society of Clinical Oncology’s clarifying statements regarding the American Society for Radiation Oncology guidelines.^1^^,^^13^^,^^14^ We chose 90 days as a practical noninitiation cut-point because it is generally not recommended in clinical practice, and it has been suggested that late RT initiation is unlikely to confer a meaningful clinical benefit.^2^ In fact, these cutoffs likely represent the outer limits of acceptable time points rather than aspirational targets. By definition, calculations based on more restrictive time points would show even lower RT initiation rates.

Indices of social risk, particularly transportation challenges and housing instability, remained strong predictors of lower RT initiation rates, even in high-density urban areas. These findings reinforce that infrastructure alone cannot overcome barriers embedded in socioeconomic disadvantage, echoing studies across breast, head and neck, and lung cancers linking social deprivation indices to delayed adjuvant therapy.^15^^,^^16^

Insurance architecture also independently influenced the initiation of RT. Medicaid and Medicare patients had substantially lower odds of timely RT initiation, a finding that persisted even when comparisons were within the same SVI strata and after adjustment for community-level risk factors. The observed payer associations should not be interpreted as direct evidence that prior authorization caused treatment delay, because those events were not measured in the available claims data. Payer type likely captures multiple administrative and care-coordination processes that may affect treatment timing, including authorization processes, utilization management restrictions, referral network restrictions, and patient out-of-pocket costs. Given that insurance type was independently predictive of lower odds of RT initiation, this interpretation is consistent with emerging data indicating that insurer-imposed administrative latency is a driver of treatment delivery disparities.^17^, ^18^, ^19^, ^20^, ^21^ In short, these findings suggest that low RT initiation rates are a symptom of systemic inefficiency and payer-specific barriers rather than social disadvantage alone, and that interventions addressing only social determinants may have limited impact without concurrent payer-level process reforms.

Our findings on these specific populations are consistent with broader trends observed in radiation oncology. Even under ideal conditions, adherence is not universal. For example, Yeboa et al^22^ reported only a 73% adherence rate in a randomized controlled trial at a leading academic center. That clinical trial setting likely enhanced adherence through close patient monitoring and rigorous protocol enforcement, conditions that are not representative of real-world care. Similarly, Graboyes et al^23^ found that 55.7% of head and neck cancer patients nationwide failed to commence postoperative RT ≤ 42 days. Our estimates for brain tumor patients are not markedly different, suggesting that difficulties with the timely initiation of adjuvant RT are a pervasive challenge in oncology care rather than one confined to a specific cancer type or institution.

When patients are at high risk of delayed RT initiation, alternative RT approaches may be medically necessary. There are existing alternative RT approaches that may mitigate the barriers identified in our study. For example, targeted RT delivered to the tumor cavity immediately following resection enables postoperative RT to be initiated immediately. Cesium-131 has been reported to be a safe and effective alternative to conventional RT.^24^^,^^25^ Similarly, preoperative stereotactic radiosurgery (SRS) may avoid the logistical challenges associated with conventional postoperative RT.^22^ However, in the case of preoperative SRS, because treatment is not delivered in conjunction with craniotomy, 100% initiation is not guaranteed. Moreover, preoperative SRS may not be clinically indicated for all tumor types.

Collectively, these findings suggest that complementary strategies are needed to comprehensively address this gap. At the health-system level, postoperative RT initiation may be improved through standardized discharge workflows, including automatic radiation oncology referrals and prospective tracking of patients who have not initiated RT within prespecified timeframes. Other disease areas, such as heart failure, have addressed analogous care gaps by developing closed-loop transition-of-care processes in which, prior to discharge, the next clinically necessary step is ordered, scheduled, assigned to an “owner,” and followed through to completion. At the payer level, delays may be reduced through streamlined authorization workflows, travel support for patients with limited access to transportation, and, when clinically appropriate, evidence-based coverage policies for the previously mentioned alternative treatment approaches, to mitigate barriers to timely RT initiation. This comprehensive approach aligns with the national academies’ call for multilevel solutions spanning infrastructure, insurance policies, and technology adoption to achieve equitable cancer care.^10^

Future research should validate risk prediction models to identify which patients are most likely to benefit from these strategies and test whether, in combination with risk prediction, these strategies improve timely RT initiation. Prospective studies should also assess whether alternative RT approaches affect the timing of systemic therapy, local control, quality of life, and overall survival.

This study has limitations. Claims data are limited by the accuracy of coding and lack potentially relevant clinical information. For example, the analytical file does not differentiate between newly diagnosed and recurrent tumors; therefore, it is possible that meaningful differences exist between these groups. This is a subject of future research we are undertaking. Because initiation dates were derived from billing codes, some misclassification of RT initiation may have occurred, for instance, in the unlikely event that RT was delivered but never billed. Moreover, claims data could also have been affected by a patient changing insurance providers between the time of craniotomy and the initiation of RT, although this is unlikely to be frequent enough to meaningfully affect the results. Clinical indication is not directly observable in claims data; as a result, a small subset may not have had RT clinically indicated, potentially biasing hospital-level RT initiation. Given that the cohort consisted exclusively of patients with malignant brain tumors, this fraction is expected to be minor. Noninitiation ≤ 90 days may have been affected by patients who died within that period or transitioned to hospice. Residual confounding by extent of resection and postoperative performance status cannot be ruled out. Proximity was indexed to the index hospital rather than to patient residence, which may under- or overestimate proximal access. Nevertheless, our use of a large national data set, the linkage to comprehensive SVI domains, and the incorporation of multiple density metrics were intended to mitigate these effects and provide a reasonable framework for evaluating how structural and social factors impact RT initiation.

Overall initiation rates of postoperative RT after craniotomy are suboptimal. The complex intersection of hospital density, social vulnerability, and payer structure underlies these persistent disparities in timely RT initiation. Addressing these factors comprehensively through regional network planning, insurance reform, and RT innovation represents an important step toward structural equity in the delivery of RT.

## Disclosures

Peter F. Orio reports consulting relationships with Theragenics, TELEFLEX, and GT Medical Technologies. Peter Nakaji reports stock and other ownership interests, as well as intellectual property relationships with GT Medical Technologies and Toragen, Inc. Tim Frandsen reports employment with GT Medical Technologies. Phillip M. Devlin reports receiving royalties from Demos Medical Publishing for a medical textbook, fees for medical-legal case review and expert consultation services, and Porosome Therapeutics stock options for service as a medical liaison.
